# Transdisciplinary Project Communication and Knowledge Sharing Experiences in Tanzania and Zambia through a One Health Lens

**DOI:** 10.3389/fpubh.2016.00010

**Published:** 2016-02-09

**Authors:** Brigitte Bagnol, Elizabeth Clarke, Mu Li, Wende Maulaga, Hilda Lumbwe, Robyn McConchie, Julia de Bruyn, Robyn Gwen Alders

**Affiliations:** ^1^Faculty of Veterinary Science, Charles Perkins Centre, University of Sydney, Sydney, NSW, Australia; ^2^Kyeema Foundation, Brisbane, QLD, Australia; ^3^Department of Anthropology, University of the Witwatersrand, Johannesburg, South Africa; ^4^Fenner School of Environment and Society, The Australian National University, Canberra, ACT, Australia; ^5^School of Public Health, University of Sydney, Sydney, NSW, Australia; ^6^Tanzania Veterinary Laboratory Agency, Dar es Salaam, Tanzania; ^7^Ministry of Fisheries and Livestock, Lusaka, Zambia; ^8^Faculty of Agriculture and Environment, University of Sydney, Sydney, NSW, Australia

**Keywords:** communication, transdisciplinarity, one health, nutrition, participatory

## Abstract

The project “Strengthening food and nutrition security through family poultry and crop integration in Tanzania and Zambia” brings together animal, crop, and human health specialists, economists, ecologists, social scientists, and practitioners to work with participating communities. It aims to increase poultry value chain, crop farming systems efficiency, and household food and nutrition security and thus requires understanding of, and ability to work effectively within, complex systems. In this context, communication knowledge sharing and synthesis between stakeholders from diverse backgrounds and a range of experiences, perspectives, agendas, and knowledge is a challenge. To address this situation, communication is conceived as a dialog and a participatory process bringing together all stakeholders. This process results in unanticipated and unexpected results that require a high degree of flexibility and adaptability from team members. The paper analyses the approach and aim of the communication strategy developed for the project and the challenges faced.

## Introduction

Improving food and nutrition security remains a global priority, requiring an integrated approach to achieve long-term, sustainable solutions. Stunting (or low height-for-age) indicates chronic restriction of growth and is associated with reduced cognitive capacity, poor school performance, lower income-earning potential, and lower birth weight of future offspring (1). Progress toward international development targets has been particularly poor in sub-Saharan Africa, where population growth has resulted in an increase in the overall number of people affected by undernutrition in recent decades (2). In Tanzania and Zambia, the prevalence of stunting in children under 5 years of age, a major determinant of individual development, is estimated to be 42 (3) and 40% (4), respectively, despite years of agricultural research and targeted health and nutrition programs.

Problems such as food and nutrition security and childhood undernutrition are regarded as “wicked” problems, meaning that they go beyond complexity and require transdisciplinary approaches. Brown (5) provides a clear and succinct definition of wicked problems that explains why problem definition and clear focus are such a challenge. “A wicked problem is a complex issue that defies complete definition, for which there can be no final solution, since any resolution generates further issues, and where solutions are not true or false or good or bad, but the best that can be done at the time” (5).

Poultry-keeping and crop systems in rural communities are highly complex, including social, economic, gender, cultural, and biophysical elements (6). Creating a cohesive and coherent research and development team and leveraging the very diverse expertise and viewpoints of stakeholders, including both male and female farmers, requires an approach to communication and evaluation that is adapted to dealing with the disorder and diversity of complex systems.

This paper documents the communication and knowledge management approach and explores learnings in a transdisciplinary research project “Strengthening food and nutrition security through family poultry and crop integration in Tanzania and Zambia,” which is addressing a current wicked problem.

## Sustainable Solutions to the Food and Nutrition Security Challenge in a One Health Approach

Efforts among human health-related multilateral agencies have historically focused on approaches such as promotion of infant and young child feeding, micronutrient fortification, and supplementation through Ministries of Health. In contrast, agriculture-related multilateral agencies have supported increased production of agricultural commodities. The lack of interconnection and the long-term sustainability of these interventions is being questioned, because many of the rural poor are not able to access fortified foods, and increased agricultural production has tended to emphasize energy-rich and nutrient-poor staples such as hybrid maize (7). There is a need for sustainable solutions that will bring the two sectors together, that is, to improve human nutrition through improved household income and dietary diversification. Local initiatives, such as enhancing traditional livestock-crop systems, can provide a sustainable solution to the ongoing demographic challenges in Africa that are driving the need for more food, improved livelihood opportunities, and reduced migration to urban centers. In addition to bringing together the two sectors, there also needs to be a deeper, more comprehensive collaboration with non-disciplinary knowledge and expertise, ranging from policy implementers and practitioners, to the beneficiaries and families themselves.

This raises the importance not only of transdisciplinary research in the sense of going beyond the disciplines to include Edmund Husserl’s “Lebenswelt” or lifeworld (8) but to a system where the whole is greater than the disciplinary parts. This requires not only disciplinary work, but “strong transdisciplinarity” with an emphasis on lack of boundaries, a consistent methodology (as opposed to methods) and a contextual and ever-changing perspective on reality (9–11).

The importance of involving a wide spectrum of disciplines in addressing complex problems such as chronic undernutrition is well-recognized (12); however, there is a need to distinguish between varying levels of integration and collaboration. Rosenfield (13) proposed a taxonomy whereby a “multidisciplinary” approach involves researchers working sequentially or in parallel within their own field to address a common problem, “interdisciplinarity” involves researchers working together but still from a disciplinary-specific basis, and “transdisciplinary” research incorporates a shared conceptual framework, which draws on various theories, concepts, and approaches.

There is considerable recognition among authors and practitioners that communication plays a crucial role in cross-disciplinary work (14) and in development projects (15–19). There are inherent issues of communication and knowledge sharing associated with transdisciplinary research. There are many differences to bridge in terms of research methods, epistemologies, work styles, assumptions as well as language (20). This is further exacerbated with the inclusion of beyond-disciplinary “Lebenswelt” stakeholders, not to mention multi-lingual and international collaborations where fundamental cultural diversities need to be bridged and included in the collective whole.

## The Project and Its Diversity

Our project “Strengthening food and nutrition security through family poultry and crop integration in Tanzania and Zambia” focuses specifically on evaluating the impact of the control of Newcastle disease (ND) in village poultry and a range of crop improvements on household food security and reducing childhood undernutrition. The project is designed to analyze and test opportunities to enhance the key role that women play in improving poultry and crop integration and efficiency to strengthen household nutrition in an ecologically sustainable manner (21).

It is a 5-year project funded by the Australian Centre for International Agricultural Research (ACIAR) and implemented by the University of Sydney (Faculty of Veterinary Science, Faculty of Agriculture and the Environment and School of Public Health) in collaboration with the Tanzanian Veterinary Laboratory Agency, the Tanzanian Ministry of Agriculture, Food Security and Cooperatives, the Tanzanian Food and Nutrition Centre, the Sokoine University of Agriculture (animal and crop health and production), Muhimbili University of Health and Allied Sciences (public health), the University of Dar es Salaam (social sciences), the Tanzanian Commission for Science and Technology, the Zambian Ministry of Fisheries and Livestock, the Zambian Ministry of Health, the National Food and Nutrition Commission of Zambia, the Tropical Diseases Research Centre, the University of Zambia (animal and crop health and production, public health and social sciences), and the Kyeema Foundation and the Royal Veterinary College in London.

This approach aspires to strong transdisciplinarity as defined above, with a strong focus on communication and synergy (as opposed to consensus). Transdisciplinarity involves crossing disciplinary and non-disciplinary knowledge boundaries to create a holistic approach. The complexity of this project requires new ways of interacting and working to transition beyond multidisciplinary and interdisciplinary approaches. This requires a greater degree of flexibility and adaptability in terms of project processes, training, dissemination, communication, including understanding the perspectives of research team members from different disciplines and practice groups, and various research approaches.

## Communication in a Complex and Transdisciplinary Project

The aims of communication processes within the context of this research project are to support the research objectives and associated outputs and facilitate the interaction between all participants and stakeholders. Communication allows those involved to “identify the attitudes, perceptions, and needs of each, and on that basis formulate explanations, recommendations and messages about policies and activities that best address the collective interest” (22).

The communication strategy in this context is a dynamic structure, requiring a number of iterations as the project unfolds. Communication processes are intended

- To ensure that the aims, objectives, and achievements of the project are well understood by key stakeholders as well as the scientific community, appropriate public institutions, and the wider community;
- To assist in the ongoing adaptive development of the project design and directions;
- To facilitate information sharing and collective knowledge creation;
- To facilitate effective, efficient, and participatory interactions within the transdisciplinary team, the broader project participants and specifically with male and female farmers;
- To foster an open and inclusive approach to different viewpoints and contributions; to open up possibilities and opportunities for multiple and ongoing solutions, and ensure these are taken into account in decision making processes;
- To create a safe space for all participants to share ideas and discuss and resolve issues in an equal and inclusive way;
- To support mechanisms for an iterative, reflective, and evaluative approach that enables ongoing learning and adaptation as well as identifying and learning from emergent ideas and strategies;
- To communicate the successes and learnings from the project to relevant stakeholders.

A transdisciplinary team and participatory approach have inherent advantages in addressing some of the challenges and opportunities of working with complex systems; however, the process is not devoid of problems. For communication in this context to be successful, there is a need to

- Create shared meanings without losing the richness of the various communities of practice with whom we are partnering;
- Build a framework for collective knowledge creation and sharing;
- Leverage different viewpoints, ways of knowing and perspectives to create a coherent whole;
- Engage with participants in design and implementation of the project;
- Accommodate a complex systems approach;
- Map the relationships between, and influence of, stakeholders;
- Learn from and consider different approaches, methodologies and viewpoints; and
- Be open to new practices and methodologies.

It is of utmost importance to facilitate the interaction with all the project participants during the project life. Emergent strategies arise throughout the process: unanticipated elements which can provide either opportunities or threats, but require an ongoing developmental evaluation process as well as a strategic communication approach (23). This process results in realized and unrealized strategies, as the project management team responds to this.

## Communication as Dialog

Brazilian educator and activist Paulo Freire’s seminal work “Pedagogy of the oppressed” (24) has had a strong influence on community development and communication. Freire developed a problem-solving approach where communication is conceived as a dialog and a participatory process for social transformation. The traditional model of communication describes a one-way linear process from sources to receivers. This top-down approach, initiated by the educated, expert or intellectual (the “haves”) and directed toward the uneducated or ignorants (the “have nots”), aims to have inform, educate, convince or persuade individuals.

In contrast, the model of communication for social change – as adopted by this project – is conceived as a horizontal, symmetrical relationship with a series of networks and nodes involving the sharing or exchange of information between two or more participants at all levels from the field (for example, the participants of this project’s randomized controlled trial) to the international level. All participants have the potential to act on the same information, none are passive receivers. The information can be created by the action of any participant or it may originate from a third source, such as a media source or religious gathering. There is an emphasis on the role of perception and interpretation of participants’ understanding, as part of a dialog or an ongoing cultural conversation. The outcomes of information processing by the participants are social perceiving, interpreting, understanding, and believing.

One important aspect in transdisciplinarity is that a broad spectrum of meanings or definitions is not only possible but essential. Early discussions within this project centered on the significance of chicken meat and eggs. Members of the research team from veterinary and public health backgrounds worked to build a shared understanding of the “quality” and “bioavailability” of protein. At the same time, there was a need to understand the widespread reluctance among farming families to eat eggs, in circumstances where chickens are scarce and represent a valuable source of cash income. Consumption of a single egg is perceived as the loss of a potential chicken. In considering the contributions of poultry to improving food and nutrition security, the aim has been to ensure that a broad, inclusive understanding of terminology is held. Rather than trying to come up with a consensus view on this, the wide variety of perspectives and knowledge is taken into account.

To advance the communication aims within the project and build strong relationships between stakeholders, regular meetings with leaders and participants at the ward and village levels are facilitated through monthly visits to the project sites by project personnel. This is intended to ensure local stakeholders remain informed and have an opportunity to contribute to project activities and share community feedback. Meetings with district leaders are also held regularly with international, Tanzanian and Zambian project personnel.

For the project to achieve significant impacts with sustainable adoption pathways, all key national (i.e., government and private sector agricultural services in addition to national agricultural research organizations) and regional stakeholders (i.e., regional economic communities and multilateral agencies) have been closely associated with the development of the project from the very early stages and throughout the project and continue to be intimately involved with its implementation. Country Coordinating Committees (CCCs), comprising stakeholders from the agriculture, livestock, and human health sectors (including representatives from government ministries, universities and other research organizations) were established in Tanzania and Zambia during the design phase and continue to meet every 3–4 months during project implementation.

The CCCs have directed the identification of project field sites (using the criteria of high stunting rates, absence of other significant nutritional interventions and contrasting agro-ecological zones) and overall national project team composition. These committees are responsible for the in-country oversight of project implementation and the communication of key findings to senior policy makers. One of the current project activities has been the development of nutrition education materials, advocating for the consumption of eggs by pregnant and breastfeeding women as well as young children. A poster, “Eat Eggs,” is being pre-tested with village residents and was discussed in the last meeting of the Tanzanian CCC in October 2015. While the general concept and text (“Eat eggs for health, strength and growth”) has been approved, feedback has been received on characteristics of images used, and the poster is currently being revised to reflect this input.

A Senior Advisory Board known as the Project Coordinating Committee (PCC) has also been established to assist with broad long-term oversight and cross-sectoral coordination. The PCC meets every 6 months, alternating between Tanzania and Zambia. The ability of research findings to contribute to positive impacts will be facilitated by undertaking the research within the regulatory, financial and policy environment in which the findings are to be applied.

The project team also coordinates and collaborates with relevant human nutrition projects and programs in Tanzania and Zambia (e.g., WHO, GAIN, UNICEF, USAID, WFP) to ensure that there are no overlapping areas of nutritional interventions and to share lessons and findings. The project uses regional institutions to provide inputs and guidance as appropriate and facilitate the sharing of lessons learnt and policy findings among member states. These regional institutions include the Food, Agriculture and National Resources Directorate of the Southern African Development Community (SADC), the East African Community (EAC), the Agriculture and Food Security Division of the African Union (AU), the Interafrican Bureau for Animal Resources (IBAR), and the Pan African Veterinary Vaccine Centre (PANVAC).

A key challenge that emerged early within the project was the lack of a complex systems focus on nutritional status in local communities. The impact of seasonal dietary fluctuations and the importance of wild foods eaten by local people had not been taken into account in previous research activities and interventions, nor had information from these various activities been shared among the organizations involved. Thirdly, dietary recommendations were not tailored to be locally and seasonally specific. To address this, an additional Small Research Activity (25) was conducted to develop locally relevant and feasible dietary diversity tools and messages. Outcomes of this project have included the development of participatory tools for: (1) collecting information about current dietary patterns, (2) suggesting optimal approaches to preparing and combining foods for people of different ages and physiological stages, (3) sharing information with communities.

There has been an ongoing focus on communication linkages and knowledge exchanges throughout the project. During a workshop held to bring together national and international institutions, it was evident that most nutrition, veterinary and agricultural specialists had not interacted and shared information in the past. Colleagues well-placed to contribute to work within this field were not always aware of the prevalence and complex causes of malnutrition within the country. The practice of consuming wild or non-cultivated foods in rural areas and the need for nutrition recommendations to be region- and season-specific have generally not been taken into consideration by those in the health sector. Collaboration with others with an understanding of local ecosystems and the seasonality of agricultural activities has the potential to contribute to a deeper understanding of the “wicked” problem of chronic undernutrition.

Participatory approaches form a central part of the diverse methodology employed by the project. Research tools such as participatory rural appraisal (PRA), participatory epidemiology (26, 27), and participatory impact assessment (28) are used regularly. Using a gender-sensitive approach, these tools have been adapted to explore the roles of men and women and address issues of access, control, and benefit over resources (29–31). These tools are also based on the notion that people learn and retain information better when their own knowledge and experience is valued, and when they are able to share and analyze their experiences in a safe collective environment. For example, during interdisciplinary field team visits, male and female farmers’ insights have been used to guide the research approach in identifying appropriate crops and crop varieties to improve human nutrition.

A significant challenge inherent in operating in diverse and complex systems is the degree of uncertainty, unpredictability, and unknowns, which arise in such a project, in part as a byproduct of the inherent “messiness” of complex, self-organizing systems. For example, long-distance travel schedules and meeting coordination are challenging to coordinate across a diversity of stakeholders with different timezones, timelines, and operating calendars (including but not restricted to the obvious differences between agricultural and academic calendars). In addition, there are several larger system variables and events, which impact on such a project, such as currency fluctuations, funding cycles, weather patterns, national and international policies, and events. Clear communication strategies and practices are essential to mitigate the impacts of these challenges. For example, the project employs a wide variety of communication approaches, including a website allowing collaborative modification by project team members, facebook page, and frequent conference calls and meetings. A number of the team members had worked together on previous projects, so these existing linkages and relationships were crucial to maintain cohesiveness within the team. Considerable time and resources are devoted to maintaining these linkages within the project with considerable benefit.

## Discussion

Achieving an enabling environment to conduct effective transdisciplinary research is a challenging and time-intensive process. Language, priorities, assumptions, experiences, methodologies and, importantly, approaches to communication can vary substantially between contributors from different disciplinary backgrounds. The slow process that a participatory approach entails is not always well understood, and its benefits not always valued. To overcome the tendency for suspicion toward unfamiliar research methodologies and results, there is a need for researchers to have confidence in the academic rigor and scientific standing of their colleagues from different fields (14). It often takes time to appreciate the value in alternative research approaches, tools, and practices. This requires an environment for team members to express their points of view and conduct open, inclusive discussions, as well as appropriate mechanisms for integration. This “safe space” depends on strong relationships, respect, trust, and frequent communication. Members of a research team need to be open to an iterative process of ongoing learning, adaptation and the creativity to deal with unplanned situations and findings.

In particular, the ability to accept and work with uncertainties and unknowns and a degree of unpredictability is the hallmark of good transdisciplinary practice. Striving for strong transdisciplinarity and research practice is an ongoing process, and the lived experience of researcher–participants operating in such transdisciplinary projects provides valuable lessons to communicate to, and share with, other transdisciplinarians.

The role of social scientists in cross-disciplinary work has been highlighted (32), as they provide illuminating insights into human behaviors and assist the different scientific disciplines to communicate more effectively. Social anthropologists’ training as acute listeners and observers means they are often aware of miscommunication before other team members and can contribute to a transdisciplinary approach (33).

Successful communication and knowledge management needs to be interwoven into the project design and implementation, not a separate area of endeavor. It should be considered an integral part of the research approach with ownership by the team in general, rather than as an optional “add on” or a separate specialist input as has often been the case in the past.

## Conclusion

Transdisciplinarity is of fundamental importance to developing sustainable solutions to complex, wicked problems. There is a need to invest time in creating an inclusive and comprehensive communication strategy to overcome challenges, allow individuals and institutions to accept unfamiliar (and at times incompatible) views and experiences, and interact effectively with colleagues from a range of disciplinary fields. It is essential that communication is an integral part of the research design and approach rather than an external input or “add on.” In addition, communication and knowledge management need to be integrated into the monitoring and evaluation planning, with clear assessment and review throughout the project. This requires commitment from not just the project team, but also from the donor agencies as well.

## Author Contributions

BB framed the paper. EC contributed with transdiciplinary research. ML added the health perspective. WM included aspect from experiences in Tanzania and HL expanded on the constraints in Zambia. RM addressed the barriers to communication. JB contributed with issues related to nutrition. RA gave an overall international perspective.

## Conflict of Interest Statement

The authors declare that the research was conducted in the absence of any commercial or financial relationships that could be construed as a potential conflict of interest.
